# Exploring hepatic stellate cell-driven fibrosis: therapeutic advances and future perspectives

**DOI:** 10.5599/admet.2874

**Published:** 2025-08-04

**Authors:** Alka Singh, Ansab Akhtar, Prashant Shukla

**Affiliations:** 1 Department of Pharmaceutical Sciences, School of Health Sciences and Technology, UPES, Dehradun, India; 2 Louisiana State University, Health Sciences Center, New Orleans, United States

**Keywords:** Retinoid receptors, non-alcoholic fatty liver disease, resmetirom, liposomes, active targeting, clinical trials

## Abstract

**Background and purpose:**

Liver fibrosis, a progressive liver disease arising from viral or metabolic causes, poses a major global health challenge due to its potential progression to cirrhosis and hepatocellular carcinoma. Due to the complex aetiology and epidemiology of liver fibrosis, most therapies fail in the clinic, and very few drugs have been approved by the US FDA.

**Approach:**

This review highlights the pathophysiological features of liver fibrosis, with a focus on novel targets in hepatic stellate cells (HSCs), key players in the fibrogenesis process, to develop successful therapeutic approaches using both pharmacological agents and active targeting strategies. The review also examines current therapeutic strategies targeting liver fibrosis, both in preclinical lab setups and clinical trials. Furthermore, various receptors involved in HSC-mediated liver fibrosis and active drug delivery targeting strategies are reviewed to enhance therapeutic outcomes. This article also integrates existing knowledge to identify research gaps and guide future investigations and clinical translation in liver fibrosis treatment. In addition, novel pathways pertaining to liver fibrosis, such as the RSPO3-LGR4/5-β-catenin cascade, the CD47/YAP/TEAD4 signalling axis, and HAb18G/CD147, are briefly elaborated in the context of therapeutic approaches for arresting HSC activation. Single-cell RNA sequencing of HSCs is presented to provide a clearer picture of liver fibrosis.

**Conclusion:**

The review highlights critical research gaps in liver fibrosis therapy and promising active targeting strategies and pharmacological interventions to improve therapeutic outcomes. Overall, this review provides a robust foundation for scientists and clinicians to advance active targeting of the disease pathology and to develop new pharmaceutical formulations that are pharmacologically safer and more efficacious.

## 1. Introduction

One out of every twenty-five deaths globally is caused by liver fibrosis, thus causing a huge health burden worldwide. Globally, the prevalence of advanced liver fibrosis in the general population was estimated to be 3.3 % (range 2.4 to 4.2 % with 95 % CI ) [1]. There are various aetiologies involved in inducing liver injuries such as alcohol, cholestasis, non-alcoholic fatty liver disease, autoimmune steatohepatitis, and drug-induced liver injury reactions [2], primary biliary cholangitis (PBC), primary sclerosing cholangitis (PSC); viral hepatitis (hepatitis B and hepatitis C); autoimmune hepatitis (AIH) [3]. These factors lead to a dynamic process of cells and tissues, responsible for extracellular matrix (ECM) deposition via the activation of hepatic stellate and myofibroblast cells.

Liver fibrosis develops as a result of the interplay of various cell types, i.e. hepatocytes, cholangiocytes, natural killer cells (NK cells), T helper cells, Kupffer cells, liver sinusoidal and epithelial cells, along with HSC. Although the main functions of hepatocytes include the clearance of hepatitis viruses, toxic metabolites, and xenobiotic compounds, as well as the secretion of lipids and proteins, apoptotic hepatocytes in an injured liver succumb, leading to the activation of myofibroblasts. In addition, inflammatory mediators, including TGF-β1, TNF-α, EGF, and IGF, are also released by injured hepatocytes and activated hepatic stellate cells, ultimately activating T cells [4], which are pivotal to both TH2-mediated fibrosis and TH17-mediated inflammation in liver fibrosis. Cholangiocytes are specialized epithelial cells lining bile ducts that play a vital role in liver regeneration, especially when hepatocyte regeneration is compromised [5]. They also activate Kupffer cells and release free radicals, TNF-α, Endothelin-1, and PDGF. Monocytes also play a very crucial role in various liver diseases, both by inflammatory functions and the resolution of inflammation. Inflammatory mediators, such as CCL2, facilitate the recruitment of LY-6C^hi^ and CCR2+ monocytes into the injured liver [6]. Collagen is produced by LY-6C+ cells, which also activate HSC and release PDGF, NKF β, CD14+, and CD16+ [7].

Liver sinusoidal and epithelial cells also have a focal role in liver fibrosis after liver injury, as these cells release endothelin-1 (ET-1) and nitric oxide (NO) [8,9]. Fenestrae, *i.e.* non-diaphragmed pores present in them, play an important role in maintaining the quiescent state of HSC and Kupffer cells, essential for a healthy liver. Natural killer cells present in the liver tissue act on pathogens and tumour substances that come through blood vessels and also play an important role in the regulation of inflammation and liver fibrosis [10]. Kupffer cells in the liver show phagocytic action towards pathogens and any hazardous substances and also perform erythrophagocytosis for senescent red blood cells. After activation, Kupffer cells release free radicals and inflammatory mediators, *i.e.* TGF-β1, TNF-α, and IGF [11]. Recently, the role of Kupffer cell pyroptosis has also been explored by researchers, demonstrating its role in promoting liver injury and inflammation, leading to liver fibrosis via the NOX2/NLRP3 inflammasome pathway [12]. In CLD associated with HBV and HCV, TH17 lymphocytes, a subgroup of T helper cells that release the pro-inflammatory IL-17A, may possess pro-fibrogenic properties. Besides, the appearance of oxidative stress, either as a consequence of excessive production of reactive oxygen species or deficiency of the antioxidant defence system, can wreak havoc in the form of liver fibrosis upon activating the HSCs. The activated HSCs initiated by oxidative damage can be detrimental, and, hence, another related phenomenon, mitochondrial dysfunction, comes into play to exacerbate the condition due to disrupted energy generation and metabolic processes, leading to apoptosis and liver damage [13].

Overall, this review presents recent and novel advancements in understanding the pathogenesis of liver fibrosis, emphasizing the major cell types involved, with a particular focus on hepatic stellate cells (HSCs). It consolidates recent clinical trial data, as well as clinical and experimental approaches that target fibrosis. Additionally, it explores delivery systems through active and passive targeting mechanisms. Lastly, future perspectives are also discussed, highlighting promising strategies for the development of effective antifibrotic therapies.

## Role of hepatic stellate cells in healthy liver and liver fibrosis

In a healthy liver, hepatic stellate cells (HSCs) play a crucial role in liver development, homeostasis, and regeneration. Vitamin A is stored by HSCs in the form of retinyl esters, located near the nucleus in the form of lipid droplets. The gut absorbs dietary retinoids, which are then carried to hepatocytes as retinyl esters and broken down into free retinol. Vitamin A is then delivered to HSCs for re-esterification, with perilipins coating these lipid droplets [14]. Adipose-differentiation-related protein is also expressed by HSCs, although its expression declines as the cells produce and shed retinoid droplets [15]. Furthermore, during liver injury, matrix remodelling is regulated by matrix metalloproteinases (MMPs) secreted by HSCs. However, tissue inhibitors of metalloproteinases (TIMPs), which are also released by activated HSCs (aHSCs), downregulate MMP activity, leading to extracellular matrix accumulation and fibrosis [16,17]. Additionally, TMPs promote the formation of collagen I in HSCs by activating TGF-β, a potent stimulant [18]. HSCs also secrete growth factors such as vascular endothelial growth factor (VEGF), which plays a key role in endothelial and epithelial cell division [19]. HSCs also express neurotrophins, including nerve growth factor (NGF), brain-derived neurotrophic factor (BDNF), and neurotrophin-3, which are important for liver function and regeneration [20,21]. Additionally, HSCs contribute to ethanol detoxification by expressing alcohol- and acetaldehyde dehydrogenases, although their role in this process is limited compared to hepatocytes [22]. Overall, in both healthy and injured livers, HSCs control ECM remodelling, play a role in tissue healing, and maintain liver homeostasis.

HSC activation is divided into two main phases: initiation, often referred to as the pre-inflammatory stage, and perpetuation. They may return to a quiescent state if liver injury is healed [23]. During the initiation phase, endothelial cells in the sinusoids play a critical role by secreting fibronectin [24] and activating latent TGF-β [25]. Platelets contribute to this process by producing TGF-β, EGF, and PDGF. Further, the latter is a potent mitogen for HSCs [26]. Additionally, various immune cells, including T cells, dendritic cells, and macrophages, interact with HSCs to modulate inflammation and fibrosis [27]. For instance, macrophages secrete TGF-β, TNF-α, MMP-9, and ROS, which contribute to HSC activation [28]. Apoptotic bodies derived from hepatocytes also play a role in stellate cell activation, without triggering an inflammatory response, and directly promote fibrogenesis [29]. Hepatocyte-expressed P450-2E1 further contributes to stellate cell activation by generating ROS during xenobiotic metabolism [30]. In translational and post-transcriptional mechanisms, including miRNAs and epigenetic regulation, stellate cell responses are modulated [31]. In the perpetuation phase, HSC activation involves cellular processes such as proliferation, fibrogenesis, chemotaxis, and matrix degradation. Upregulation of PDGF receptor expression via TNF-α enhances the fibrogenic phenotype of stellate cells [32]. Matrix metalloproteinases (MMPs), such as MMP-2 and MMP-9, play dual roles in fibrosis progression; while they regulate ECM turnover, their antifibrotic expression declines as fibrosis advances [33]. Moreover, stellate cells also express a variety of chemokine receptors that are important in cell migration and subsequent activation, including CXCR4 and CCR5 [34,35]. TGF-β1 stimulates ECM production through autocrine and paracrine pathways, while connective tissue growth factor (CTGF) amplifies profibrotic signals via a G-protein-coupled receptor [36]. Micro iRNAs, especially miR-29b, play a major function in regulating collagen synthesis via inhibiting TGF-β-induced collagen production [37]. Crucially, mitochondrial ROS and HIF-1α-mediated hypoxia-induced signalling pathways, such as ERK1/2 and JNK1/2, further promote HSC migration and activation, underscoring the intricate interactions of molecular pathways implicated in liver fibrosis [38]. Regulatory T cells (Tregs) mitigate hepatic fibrogenesis by producing IL-10, which inhibits HSC activation and the production of TH17-derived IL-17, while also limiting Kupffer cell activation [39].

**Figure 1.**
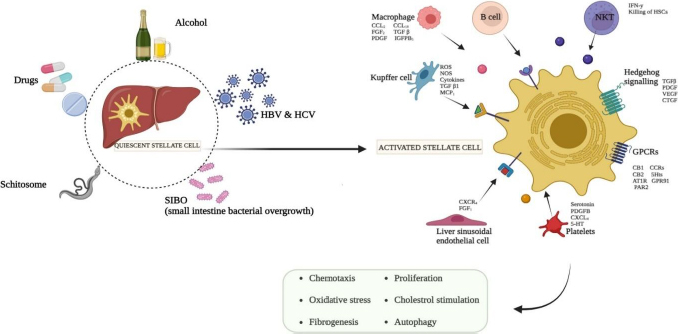
Pathophysiology of hepatic stellate cells. Several dramatic changes occurred during the development of liver fibrosis at cellular level. Liver fibrosis is incurred by several factors, including drugs, schistosomes, SIBO, HCV & HBV, and alcohol followed by HSC activation. Regular injury fosters a chronic liver disease. In activated HSC cells, collagen and extracellular matrix accumulation, vitamin A is lost, and oxidative stress occurs. Overall, cells including Macrophages, Kupffer cells, platelets, B cells, and natural killer cells are involved in the activation of HSC

Although initially believed to play protective roles in NAFLD, mucosal-associated invariant T cells (MAIT) depletion in mice has been connected to fibrosis resistance. More recently, it has been demonstrated that MAIT cells induce fibrosis by stimulating HSCs through IL-17 and macrophage pro-inflammatory polarization [40,41]. Similarly, γδ T cells, which accumulate in fibrotic livers, can induce HSC apoptosis and enhance natural killer cell-mediated cytotoxicity, thereby limiting fibrosis, though they also contribute to IL-17 production, supporting fibrogenesis [42,43]. Innate lymphoid cells (ILCs), particularly NK cells, play a pivotal role in liver fibrosis, with NK cells exhibiting antifibrotic effects by killing activated HSCs and releasing IFNγ, offering a potential therapeutic strategy by boosting their activity [44,45]The intricate relationships among these many immune cell types highlight the complexity of liver fibrosis, where the resolution or development of the illness is determined by the balance between profibrotic and antifibrotic signalling pathways (see Figure 1).

### The RSPO3-LGR4/5 axis in quiescent hepatic stellate cells

Recently, Sugimoto *et. al.* [46] demonstrated that R-spondin 3 (RSPO3), secreted by quiescent HSCs, plays a crucial role in supporting hepatocyte metabolism, regeneration, and survival. These findings are contrary to the traditional view of HSCs functioning solely as fibrogenic drivers in chronic liver disease (CLD). The study demonstrated that as CLD progresses, RSPO3 expression declines, leading to worsened liver function and fibrosis. The RSPO3-LGR4/5-β-catenin pathway was demonstrated as a crucial communication axis between HSCs, hepatocytes, and endothelial cells, coordinating liver zonation and function. Therapeutically, restoring RSPO3 or reverting HSCs to a quiescent state may simultaneously suppress fibrosis and restore liver function, offering a promising strategy for treating diseases like MASLD and ALD [46].

### Single-cell RNA sequencing of hepatic stellate cells in understanding liver fibrosis

A very recently reported study [47] has successfully presented the first single-cell atlas of HSCs across seven liver injury models in mice (using 10 experimental single-cell sequencing data), revealing a conserved HSC activation trajectory that is also preserved in human livers. The group identified three distinct HSC subtypes—quiescent (qHSCs), initiatory, and myofibroblasts— across all injury types, suggesting a universal activation mechanism for liver fibrosis. The outcome of the reported study demonstrated key transcription factors (Rxra, Foxf1, Nr1h4 for qHSCs; Fosl1, Egr3, Nfkb2 for initiatory HSCs; and Wt1, Prrx1, Mef2c for myofibroblasts) and ligands (TGF-β, EGF, SPP1, AGT, and parathyroid hormone) functioning as conserved drivers of HSC activation.

The authors successfully identified [47] and reported COLEC10 as a highly specific HSC marker and potential biomarker for liver fibrosis, outperforming traditional markers such as FIB4 and APRI. This report has paved the way further for the potential for broad application of anti-fibrotic therapies targeting conserved TFs or ligands and markers for their potential role in preclinical drug discovery for liver-related injuries [47].

### CD47YAP/TEAD4 pathway in hepatic stellate cells activation

There are additional novel signalling cascades involved in HSC activation, one of which is CD47, a YAP target gene and transcriptional factor that plays a role in cell proliferation, development, and survival. CD47 is also known to prevent phagocytosis by inhibiting macrophage activity, and TEAD4 has a developmental role. Here, YAP and TEAD4 function in a complementary manner to each other in the induction of CD47 transcriptional activation. This pathway contributes to the activation of HSC, and hence, liver fibrosis can be propagated. The CD47/YAP/TEAD4 signalling axis is more inclined to the association of non-alcoholic fatty liver disease (NAFLD). Upregulated CD47, triggered by the YAP and TEAD4 genes, leads to the heightened activation of HSCs, resulting in liver fibrosis. Therefore, silencing or knocking down the CD47/YAP/TEAD4 pathway can inhibit the fibrotic gene expression, thereby attenuating liver fibrosis pathology. In this context, anti-CD47 antibodies have been investigated for their potential to alleviate fibrosis-associated inflammation. Moreover, pharmacological intervention in the communication halting between YAP and TEAD4 will further potentially reduce the chances of HSC activation and liver fibrosis [48].

### HAb18G/CD147 role in hepatic stellate cell activation

Activation of the HAb18G/CD147 pathway has been reported to stimulate HSCs and induce the overexpression of fibrogenesis-associated genes. The HAb18G/CD147 protein is expressed on the surfaces of HSCs in cases of liver fibrosis. This phenomenon arises due to extracellular matrix remodelling and collagen production, which are responsible for liver fibrosis. As a therapeutic target, antibodies have been investigated in preclinical animal studies and found to be effective in downregulating the HAb18G/CD147 cascade, thereby alleviating HSC-activated liver fibrosis [49].

## Therapeutic approaches

### Drugs approved by the US-FDA and in clinical trials

**Figure 2.**
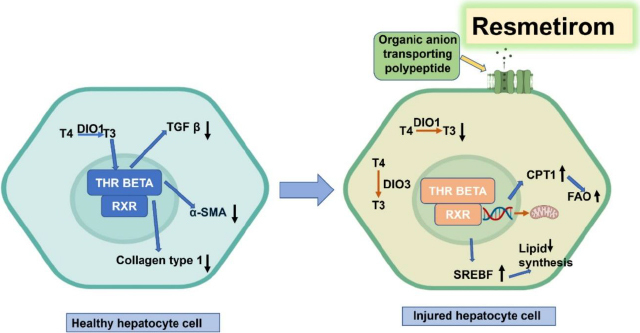
Mechanisms of action of thyroid hormones and resmetirom in regulation of hepatocyte lipid metabolism., Thyroxine (T4), Iodothyronine deiodinase 1 (DIO1) Triiodothyronine (T3), Nuclear thyroid receptor β (THR-β), Retinoid X receptor (RXR) Carnitine palmitoyltransferase I (CPT1), Mitochondrial fatty acid oxidation (FAO), and Sterol regulatory element binding transcription factor 1 (SREBF1) Iodothyronine deiodinase 1 (DIO3), reverse T3 (rT3). Modified from [57], Creative Commons CC BY

**Figure 3.**
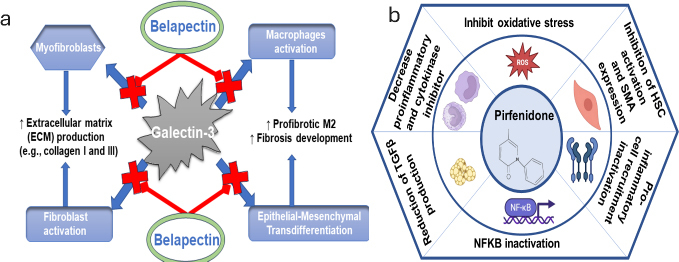
a - targets of belapectin for alleviation of fibrosis; b - Molecular mechanisms of action of pirfenidone. It affects signalling pathways such as the Smad pathway, which is downstream of TGF-β, leading to a decrease of fibrogenic activity. Consequently, it inhibits fibroblast proliferation and increases fibroblast apoptosis

Extensive research has been conducted on various pharmacological methods to combat liver fibrosis, to address the fundamental processes driving fibrosis and to develop effective therapies. One of the most widely explored targets is PPARα and PPARδ. In a recently reported study [50], the activation of PPARα and PPARδ has been observed to alter metabolic pathways and decrease inflammation, both of which are important elements in the development of liver fibrosis. It has been demonstrated that elafibranor effectively enhances markers for liver fibrosis and slows down the progression of the condition in a non-alcoholic steatohepatitis animal model [50]. Pioglitazone, another PPARγ agonist, has also been investigated for its ability to treat liver fibrosis. Huang and colleagues revealed that pioglitazone enhanced liver histology and lowered the expression of fibrogenic genes in an animal model of non-alcoholic steatohepatitis [51]. The FXR, a nuclear receptor crucial for maintaining bile acid equilibrium and regulating liver metabolism, is strongly and selectively stimulated by tropifexor. Furthermore, some researchers observed that tropifexor decreases liver fibrosis and enhances various indicators of liver well-being in an animal model with non-alcoholic fatty liver disease [52]. The researchers propose that emricasan's anti-fibrotic effects result from its ability to inhibit caspase-mediated apoptosis and inflammation, both of which play crucial roles in the development of liver fibrosis [53]. To treat this disease, several medications have been examined, which are discussed in Table 1. Recently, the FDA approved resmetirom for non-cirrhotic MASH with moderate to advanced fibrosis, based on a Phase 3 clinical trial (NCT03900429), which demonstrated MASH resolution with at least one stage improvement in fibrosis [54]. This drug acts by targeting the thyroid hormone receptor (THR)-β, which is present in the liver. The detailed molecular mechanism of the effects is presented in Figure 2. Pirfenidone is an antifibrogenic and anti-inflammatory drug and is approved by the US-FDA for idiopathic pulmonary fibrosis and is currently under investigation for liver fibrosis and various other fibrotic diseases. It acts by affecting multiple molecular targets, notably inhibiting TGF-β and reducing pro-inflammatory cytokines, leading to alleviated fibrosis (Figure 3b). Its hydroxy analog, hydronidone, has been proven better for the therapy of liver fibrosis owing to its high hepatic safety (due to metabolism by Phase 2 transformation instead of Phase I transformation).

**Table 1.** Drugs/compounds used in clinics to treat liver fibrosis

Name	Original indication	Target	Current status / Effects	Ref.
Belapectin	Liver fibrosis	Galectin-3 antagonist	NCT02462967 phase 2b: NCT04365868 phase 2/3 prevention of oesophageal varices	[58,59]
Saroglitazar	Diabetic dyslipidaemia	PPARα/γ agonist	Phase 2	[60]
Elafibranor	Primary biliary cholangitis	PPARα and PPARδ agonist	Not effective phase 3	[61]
Seladelpar	Primary biliary cholangitis	PPARδ agonist	Accelerated approval US-FDA	[61]
Resmetirom Rezdiffra™	NASH with Fibrosis	THR-β agonist	Accelerated approval US-FDA	[62]
Liraglutide	Diabetes and obesity	GLP-1 analog	Diabetes associated liver fibrosis	[63]
Semaglutide			Metabolic disorders associated steatohepatitis linked liver fibrosis	[64]
Obeticholic acid	Liver fibrosis	FXR agonists	Accelerated approval US-FDA currently restricted use	[65]
Setanaxib	Liver fibrosis	NOX1, NOX4 inhibitor, NADPH oxidase	Fast track approval, FDA (Orphan)	[66]
Pentoxifylline	Peripheral vascular disease	Non-specific phosphodiesterase inhibitor	Benefits in small clinical trials not approved by US-FDA	[67]
Hydronidone	NA	Wnt/β-Catenin pathway inhibitor	Demonstrated efficacy in phase 2 and phase 3 trials	[68]
Fluorofenidone	NA	Inhibited TGFβ1/Smad and MAPK signalling	Limited clinical trial data	[69]
Tirzepatide	Type 2 diabetes	GLP-1 and GIP agonist	Phase 2 resolution of MASH without worsening liver fibrosis	[70]
GS-0976 / Firsocostat	NA	Acetyl-CoA carboxylase (ACC)	Phase 2 NASH	[71]
Pegbelfermin	NA	FGF21 analogue	Failed Phase 2b	[72]
Aldafermin	NA	FGF19 analogue	Phase 2/3 promising results	[73]
Aramchol	Metabolic disorders	Inhibitor of de novo lipid synthesis	Effective in Phase 3 ARMOR study	[74]

Additionally, the drug leads to enhanced Smad 7 upregulation and binding to TGFβRI, resulting in the inhibition of phosphorylation of profibrotic Smad 2/3 and subsequent degradation of TGFβRI [55]. Furthermore, belapectin is a carbohydrate-based drug developed by galectin Therapeutics that acts on the molecular level by inhibiting the effects of Galectin-3 on various cells (see Figure 3a), leading to reduced extracellular matrix deposition and fibrosis development [56].

## Experimental approaches

Recent studies have demonstrated the potential for improving the treatment approaches through the utilization of specific targeting agents and ligands, which focus on particular markers expressed on target cells. Targeted delivery methods have demonstrated encouraging outcomes in enhancing the precision and effectiveness of therapeutic approaches [75]. The receptors playing an important role in liver fibrosis and emerged as potential targets for the therapy of liver fibrosis include nuclear receptors, *i.e.* liver X receptors (LXR), farnesoid X receptor (FXR), retinoid X receptor (RXR), vitamin D receptor (VDR), cannabinoid receptors, Toll-like receptor (TLR), and peroxisome proliferator-activated receptors (PPARs) (See Table 2).

**Table 2.** Experimental approaches of the therapy of liver fibrosis

Target	Drug/components	Function/pharmacological implication	Ref.
FXR	ID119031166 (ID166)	Regulate bile acid homeostasis and ↓ liver fibrosis and NASH pathology without activating itch-related receptors	[86]
	GW4064	Inhibition of NF-KB and decrease cytokine-STAT3 signalling	[87]
	INT-787 and OCA	Modulate RECK expression, ↓ MMPs, ADAM10, and ADAM17 activity	[88]
	INT-767, OCA	↓ of collagen and induced MMP2-9 activity	[89]
	GCDCA	Activating the NLRP3 inflammasome pathway	[90]
LXR and FXR	Acanthoic Acid	↓lipid accumulation, ↓SREBP1, ↑AMPK-SIRT1 signaling	[76]
LXR	Sesamol	Inhibits autophagy markers MAPLC3α/β, P62	[91]
	SR9238	SR9238 inhibits the activity of LXR	[92]
PPARγ agonist	GSK2033	Inhibit activity of LXR	[93]
PPAR agonist	Ligustrazine	Inhibited HSC migration, adhesion, contraction, and angiogenic cytokine production	[94]
PPARα	Methoxyeugenol	Inhibiting HSC activation	[95]
	Oleoylethanolamide	Block activation of HSC inhibits alpha smooth muscle actin, TGF-β1	[96]
	Pirfenidone	Induced PPAR-α/carnitine O-palmitoyltransferase 1/acyl-CoA oxidase 1	[97]
PPARα / δ agonist	ZLY16	Regulating gene expression including COLIA1, TIMP, TGFβ, TNFα and IL6	[98]
PPARα / γ agonist	Saroglitazar	Decrease liver collagen level and cholangiocyte proliferation marker (CK9)	[99]
Integrin Subunit Alpha 11 (ITGA11)	MicroRNAs (Eugol)	miR-12135 suppressed Integrin Subunit Alpha 11 (ITGA11) likewise suppress fibrosis	[100]
PDGFRβ	Crenolanib	Crenolanib inhibit PDGFR-β inhibit HSC proliferation	[101]
	Salvianolic acid B	Inhibit HSC activation migration and proliferation	[102]
PDGF	Benzimidazoisoquinoline derivatives	↓ PDGF and TGFβ-induced proliferation	[103]
PDGFRβ	Gomisin D	Inhibit HSC proliferation, activation and promote apoptosis	[104]
IGFIIR	Aptamer-20 (SELEX)	High-affinity ligand to receptor for antifibrotic activity	[105]
Cannabinoid receptor	AM1241	Inhibits the TLR4/NF-κB pathway	[106]
	Cannabigerol	↓ hepatic inflammation and fibrosis	[107]
	Exosomes	Upregulating CB1 receptors and downregulating CB2 receptors	[108]
CB1 receptors	JD5037	Activated HSCs via β-arrestin1/Akt signalling, which is inhibited by JD5037	[81]
CB2 receptors	AM1241	Inhibiting the TLR4/miR-155/NFκB p65 pathway	[82]
Toll like receptor	ALT-100	Neutralizing eNAMPT, thereby inhibiting the eNAMPT/TLR4 inflammatory pathway	[84]
	Rutin	Inhibiting the TLR4 (Toll-like receptor 4) and P2X7r signalling pathways	[109]
Protein tyrosine phosphatase 1B	Luteolin-7-diglucuronide	↓ TGF-β1-mediated overexpression of collagen 1, α-SMA and fibronectin	[110]
NF-κB/NLRP3 inflammasome signaling axis	Mangiferin	↓NF-κB/NLRP3 inflammasome pathway, ↓ α-SMA hepatic expression and fibrous tissue deposit. ↓ MDA and ↑ GSH and Nrf2	[111]
Apolipoprotein L2	12-deoxyphorbol 13-palmitate	↓APOL2-SERCA2-PERK-HES1 signalling	[112]
GSK-3β modulation	Fluorofenidone	Targets GSK-3β/β-catenin pathway, ↓ β-catenin-mediated pro-fibrotic gene expression, ↑ Nrf2/HO-1 pathway ↓ oxidative stress, ↓ hepatocyte apoptosis	[113]
AMPK-PPARγ pathway	Arctigenin	↓ expression of fibrosis-related genes (Col1a1, Col3a1, Acta2), reversal of activated HSCs to quiescent HSCs	[114]

LXR belongs to the family of nuclear receptors and regulates the accumulation of fat, the level of inflammation, and scarring of liver tissue with fibrosis in NAFLD. LXRα expression was positively correlated with intrahepatic expression of CD36, SREBP-1c, and ABCG5/8. They demonstrated that LXR is an attractive therapeutic target for liver fibrosis. GW3965 is an agonist of LXR and reduces signs of scarring and inflammation, as well as the activation of hepatic stellate cells in mice [76].

FXR is a bile acid-activated nuclear receptor, also known as NR1H4. FXR contributes to preventing HSC activation and retaining their quiescent state. FXR lowers the risk of bile acid-induced liver damage by controlling the expression of genes related to bile acid metabolism. FXR maintains quiescent HSCs and controls bile acid levels, so it plays an important role in controlling liver fibrosis [77,78].

RXR is involved in several biological processes, including the regulation of transcription factors and gene expression. RXR-α, RXR-β, and RXR-γ are the three main types of retinoid X receptor. Ligands activate RXRs, like 9-cis retinoic acid, after activation modulates transcription of genes that control cell proliferation, differentiation, and death, as well as maintain HSCs in their quiescent state. Also, it has been reported that vitamin A (VA) decorated valsartan-loaded liposome therapy (VLC) leads to the re-expression of PPAR-γ, with a significant reduction in fibrogenic mediators, and nearly normalized liver function tests. These liposomes were coupled with VA to specifically target HSCs [79,80].

Cannabinoid receptors (G protein-coupled receptors) are part of the endocannabinoid system, having two subtypes, namely CBR1 (present in hepatocytes) and CBR2 (immune cells present in the liver). Activation of CBR1 has been associated with lipid accumulation in hepatocytes, leading to liver fibrosis [81]. CBR2 displays protective behaviour against liver fibrosis through reducing inflammation and regulating immune response [82].

Likewise, Toll-like receptors are a class of proteins that control the activity of the innate immune system. By focusing on pathogen-associated molecular patterns (PAMPs) and damage-associated molecular patterns (DAMPs), they elicit an immune response. In this context, it has been observed that HSCs activation also gets upregulated by TLR4 signalling and promotes fibrosis by releasing profibrotic factors and pro-inflammatory cytokines [83]. Scientists also reported [84] that controlling TLR signalling is a potential therapeutic approach for preventing the development of liver fibrosis. ALT-100, a monoclonal antibody that neutralizes eNAMPT, significantly reduces NAFLD severity and progression to NASH/hepatic fibrosis by attenuating liver inflamemation, triglyceride accumulation, and fibrosis in both human and mouse models. These results suggested that targeting the eNAMPT/TLR4 pathway could be a promising therapeutic strategy for NAFLD [84].

Furthermore, PPARs (subtypes: PPARα, PPARβ/δ, and PPARγ) agonists have emerged as an important therapeutic option for liver and other metabolic disorders by boosting antioxidant factors and reducing inflammation. PPARα, which regulates lipid metabolism and inflammation and has an indirect role in the activation of HSCs, is an important target for preventing the progression of liver fibrosis.

PPARγ is important in reducing liver fibrosis because it has the potential to inhibit the activation of hepatic stellate cells, regulate lipid metabolism, and reduce inflammation and tumour growth in the liver. On the other hand, the role of PPARβ/δ in preventing liver fibrosis was demonstrated by Zang *et al.* [85], uncovering a new mechanism by which a PPARβ/δ agonist (GW501516) decreases TGF-β1-led activation of HSCs and fibrosis via AMPK signalling, leading to a reduction in both SMAD3 phosphorylation and p300 levels.

## Drug delivery in liver fibrosis

The liver, as an organ, is actively involved in the metabolism of drugs, and most drugs encounter liver tissue at higher concentrations due to the presence of receptors and transporters in hepatocytes. Apart from the presence of a major macrophage population, it also facilitates the accumulation of high-molecular-weight drugs, especially peptides and proteins. However, in the case of liver fibrosis, the involvement of other cell types present in the liver has been demonstrated; hence, targeting cells apart from hepatocytes and macrophages has become an important approach that has been explored recently for therapeutic intervention in liver fibrosis.

### Active targeting

**Figure 4.**
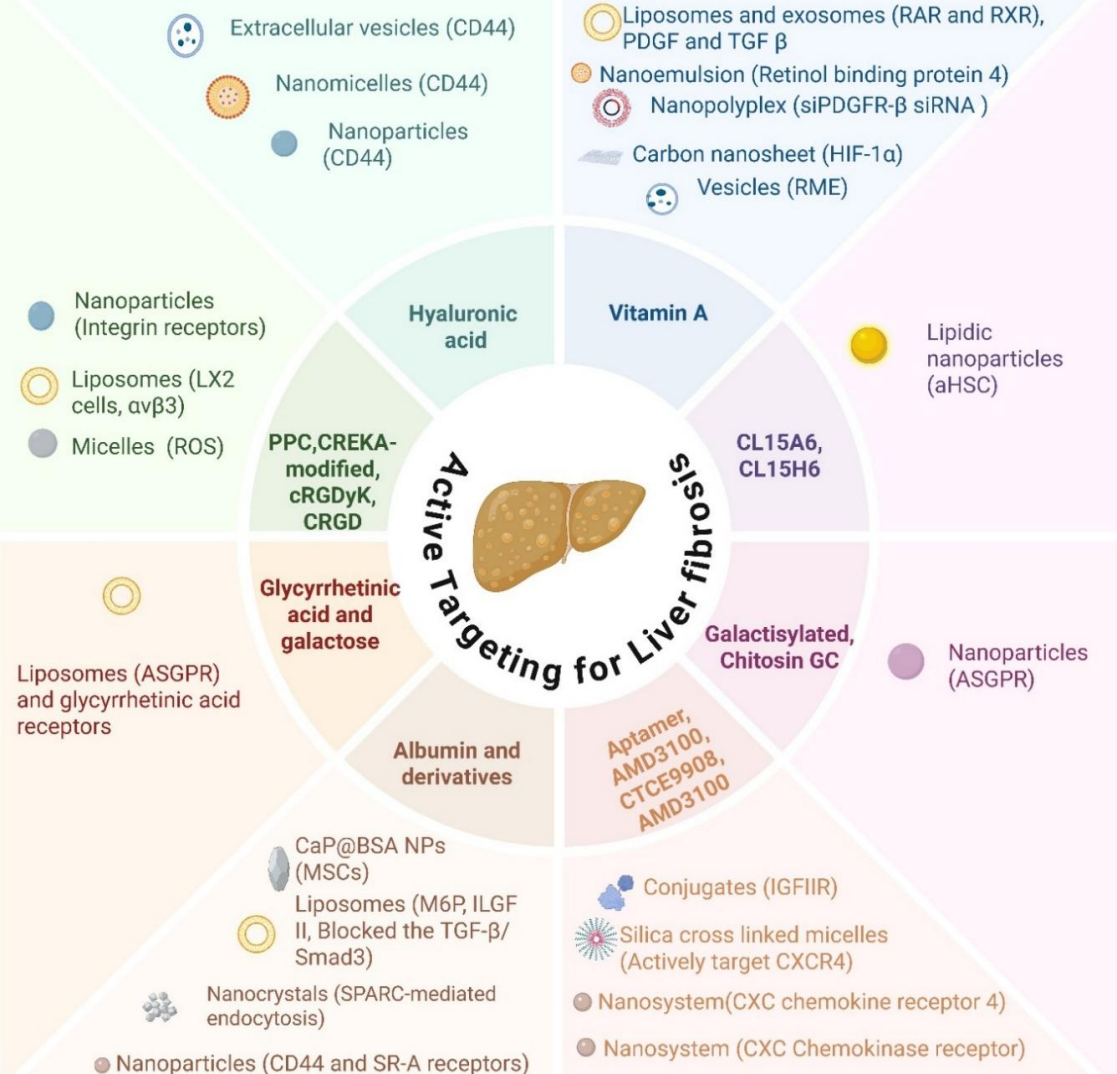
Major drug delivery systems with active targeting agents use for the therapy of liver fibrosis (Created in BioRender. Awasthi, R. (2025) https://BioRender.com/obrtwsc)

Targeted delivery of drugs to specific cell subpopulations in the liver can be a crucial strategy, particularly with a deeper understanding of the function of specific cell subtypes in liver fibrosis. Even in the same organ, this method reduces undesirable side effects and improves the therapeutic outcome of the treatment. Cell-specific targeting employs specific ligands targeting receptors selectively expressed in the desired cell type, in the case of a disease, in addition to the physicochemical properties of nanocarriers. These ligands are attached chemically to the nanocarrier surface, enabling active targeting and increasing the specificity of the therapy. A summary overview of active targeting drug delivery systems and their associated ligands in liver fibrosis is presented in Figure 4.

Recent studies demonstrated utilization of innovative nanocarriers for targeting hepatic stellate cells (HSCs) in liver fibrosis. Many carriers, such as silica cross-linked micelles coloaded with silibinin and sorafenib modified with the CTCE9908 peptide, CD44 receptor-targeted nanoparticles co-loaded with probucol and collagenase I, palmitic acid-modified albumin nanoparticles, and Endothelin receptor antagonist (CH948, a derivative of PD-156707) conjugated SPIONS MRI, have also been explored (Table 3).

**Table 3.** Experimental approaches for active targeting of liver fibrosis

Molecular targets/receptors	Targeting ligand	Carrier	Drug	Outcomes	Ref.
PDGFR-β / RARs	Vitamin A (VA)	Nanopolyplex	siPDGFR-β siRNA	Inhibited HSC activation; ↓ pro-fibrogenic proteins and hepatic collagen; restored liver function in mice	[120]
ECM	Collagenase	Chitosan nanoparticles	siRNA	Enhanced liver uptake and cell-specific delivery with collagenase pre-treatment	[121]
aHSC	CL15A6, CL15H6	Lipid nanoparticles	siRNA cocktail	Knockdown of Hh and TGFβ1 reversed fibrosis; reprogrammed aHSCs to qHSCs	[122]
HIF-1α+ HSCs	VA-PEG	CN@GQDs nanosheets	HIF-1α siRNA	TGF-β1/Smad inhibition; effective siRNA delivery under hypoxia	[123]
CXCR4	CTCE9908 peptide	Silica micelles	Silibinin + Sorafenib	↓ Inflammation/collagen; ↓ HSC proliferation; ↑ apoptosis.	[124]
IGFIIR	Aptamer	Conjugate	siRNA	Diagnostic and therapeutic tool with high affinity	[106]
CD44 CD44 RBPR	Hyaluronic acid Retinoic acid cRGDyK	Nanoparticle	Curcumin	Promising antifibrotic effect via HA-PLA NPs.	[125]
		Nanomicelles	Silibinin	Strong antifibrotic effects in HA-based nanomicelles	[126]
		Nanoparticles	Galangin	↑ Solubility; anti-fibrotic via RA targeting	[127]
αvβ3 integrin		Liposomes	Vismodegib	Targeted delivery to aHSCs over qHSCs	[128]
LX-2 cells	CREKA peptide	Liposomes	Sorafenib	Reduced ECM and HSC activation	[129]
RARs	Vitamin A	Cationic liposomes	TLR4 shRNA	Effective aHSC targeting; antifibrotic outcome	[130]
PDGF + TGFβ	Vitamin A	Liposomes	Imatinib	Better antifibrotic effects with VA-conjugation	[131]
ROS	cRGD	Micelles	Resveratrol	ROS-responsive release in aHSCs	[132]
M6P/IGF-II receptor	M6P-albumin	Liposomes	Hesperidin	↑ Targeting; ↓ required dose.	[133]
GA receptor + CD44	GA + HA	Liposomes	Curcumin + Berberine	Synergistic anti-aHSC and pro-apoptotic effect	[134]
RARs + RXRs	Vitamin A	Liposomes + exosomes	Hydroxychloroquine	Hybrid system effectively targets fibrosis	[135]
CXCR4	AMD3100	Liposomes	Pirfenidone	↓ CXCR4, TGFβ, α-SMA; effective antifibrotic response	[136]
HSCs SPARC receptor	Collagenase I + retinol albumin	Nanodrill micelles	Nilotinib	Deep tissue penetration; strong antifibrotic effect	[80]
		Nanocrystals	Silibinin	High bioavailability; PDI < 0.15; ~60 nm	[137]
PDGFR-β / RARs	Vitamin A (VA)	Nanopolyplex	siPDGFR-β siRNA	Inhibited HSC activation; ↓ pro-fibrogenic proteins and hepatic collagen; restored liver function in mice	[138]
ECM	Collagenase	Chitosan nanoparticles	siRNA	Enhanced liver uptake and cell-specific delivery with collagenase pre-treatment	[139]
aHSC	CL15A6, CL15H6	Lipid nanoparticles	siRNA cocktail	Knockdown of Hh and TGFβ1; reversed fibrosis; reprogrammed aHSCs to qHSCs	[140]
HIF-1α+ HSCs	VA-PEG	CN@GQDs nanosheets	HIF-1α siRNA	TGF-β1/Smad inhibition; effective siRNA delivery under hypoxia	[141]
SPARC receptor	Albumin	Albumin coated liposomes	Naringenin	better antifibrotic effects in mouse model	[142]
IGFIIR	Aptamer	Conjugate	siRNA	Diagnostic and therapeutic tool with high affinity	[143]
CD44	Hyaluronic acid	Nanoparticle	Curcumin	Promising antifibrotic effect via HA-PLA NPs	[79]
CD44	Hyaluronic acid	Nanomicelles	Silibinin	Strong antifibrotic effects in HA-based nanomicelles	[144]
RBPR	Retinoic acid	Nanoparticles	Galangin	↑ Solubility; anti-fibrotic via RA targeting	[145]
αvβ3 integrin	cRGDyK	Liposomes	Vismodegib	Targeted delivery to aHSCs over qHSCs.	[146]
LX-2 cells RARs	CREKA peptide	Liposomes	Sorafenib	Reduced ECM and HSC activation	[124]
	Vitamin A	Cationic liposomes	TLR4 shRNA	Effective aHSC targeting; antifibrotic outcome	[147]
PDGF + TGFβ	Vitamin A	Liposomes	Imatinib	Better antifibrotic effects with VA-conjugation	[148]
ROS	cRGD	Micelles	Resveratrol	ROS-responsive release in aHSCs.	[149]
M6P/IGF-II receptor	M6P-albumin	Liposomes	Hesperidin	↑ Targeting; ↓ required dose.	[150]
GA receptor + CD44	GA + HA	Liposomes	Curcumin + Berberine	Synergistic anti-aHSC and pro-apoptotic effect	[151]
Asialoglycoprotein receptor + GA receptor	Galactose-PEG2000-NH_2_ (GalNH_2_) + Glycyrrhetinic acid	Nanostructured Lipid Carriers	Dehydrocavidine	↑ liver targeting, ↑ uptake by aHSCs, apoptosis induction ↓fibrosis markers	[152]
Cation-independent mannose-6-phosphate receptor (CI-M6PR)	Ligands targeting TGF-β Receptor I and CI-M6PR	Polydopamine (PDA) Nanoparticles	Ligand targeting TGF-β Receptor I	↑Accumulation in fibrotic liver tissue ↓ ROS ↓Transforming growth factor-beta (TGF-β) production targeted lysosomal degradation of TGF-β Inhibition of TGF-β-Smad2/3 signaling pathway ↓ECM secretion Significant attenuation of liver fibrosis	[153]
Collagen I (ECM component)	Succinylated Gelatin	Succinylated gelatin -coated Liposomes (SG-lip)	Pirfenidone	Prolonged retention, enzyme-responsive release, ↓ fibroblast proliferation, invasion, and myofibroblast differentiation	[154]
CD44	Chondroitin Sulfate-Hexadecylamine conjugate (CS-HDA)	Amphiphilic CS-HDA-based nanoparticles	Imatinib	↑ uptake by aHSCs; fibrosis resolution in mouse model	[155]
Asialoglycoprotein receptor	DSPE-PEG-galactose	Self-assembled SHG nanoparticles	Sorafenib and hederagenin derivative (Hed)	↓ Collagen deposition by 57.5 ± 2.3% (vs. 24.8 ± 1.8% with sorafenib alone); ↑ uptake in ASGPR-overexpressing HSCs; ↑ anti-fibrotic efficacy (in vivo)	[156]
Retinol-binding protein receptor (RBPR)	Vitamin A (VA)	Vitamin A-PEG-PNTC(poly(ethylene glycol)-poly(nitrate carbonate) Micelle	Benazepril (BN)	Targeted delivery to activated HSCs; controlled NO release led to ↓ α-SMA, induction of apoptosis, and ↓ fibrosis	[157]
TGF-β1	Galactose	Bilosomes	Sofosbuvir	Drug loaded bilosomes improved liver function (↓ AST, ALT, ALP), ↓ TGF-β1 levels, and regressed liver fibrosis score in animal model	[158]
α-SMA, COL1A1	IGFBP-3-targeting moiety	Xanthosomes	TMEM219 (Transmembrane protein 219)	↓ α-SMA, ↓ COL1A1, ↓ IGFBP-3, ↓ TGF-β1, ↓ p-AKT; ↓ALT, AST); ↓ necrosis, inflammation, and fibrogenesis in BDL-induced fibrosis	[159]
FAP-α	Hepatic fibrosis-targeting peptide (FAP-α-responsive)	FAP-α- nano shells (PFD@ns)	Pirfenidone (PFD)	Targeted delivery to fibrotic liver, ↓ TGF-β1-induced collagen production, ↓ HSC activation, ↓ inflammation, modulation of PI3K/AKT/mTOR pathway, ↑ antifibrotic efficacy, ↓ off-target effects	[160]
Retinoid receptors	Retinoid (Vitamin A)	Retinoid decorated RBC membrane encapsulated polydopamine nanoparticles	Sorafenib	↑ aHSC targeting, ↓ HIF-1α & glycolysis, reversal of myofibroblast phenotype, ↓ liver inflammation and fibrosis in CCl_4_-induced mice	[161]
HA receptor (*e.g.* CD44 on activated HSCs)	Hyaluronic acid (HA)	Cu^2+^-coordinated mesoporous	Curcumin	Effective liver targeting, ROS scavenging, ↓ HSC proliferation, collagen deposition, ↑ antioxidant activity, pH-responsive drug release, and alleviation of liver fibrosis	[162]

### Advances in active targeting

Inherent redundancy in fibrogenic signalling pathways means that targeting a single molecular pathway often fails to halt disease progression. This fact has recently led to the development of various experimental therapeutic approaches targeting multiple targets using a combination of two or more drugs and targeting approaches (see Table 3).

Recently, a reported study demonstrated a novel targeted therapeutic strategy for liver fibrosis using mesenchymal stromal cells (MSCs) engineered with a high-affinity peptide ligand (pPB) specific for the platelet-derived growth factor receptor beta (PDGFRβ), which is overexpressed on activated hepatic stellate cells (aHSCs). This target was identified through an analysis of previously published differential cell expression data and single-cell RNA expression data, utilising *in silico* methods [116]. The investigators successfully functionalized MSC membranes [116] with the pPB peptide via hydrophobic insertion, a rapid and clinically amenable technique that preserves viability, cellular membrane integrity, and functionality. Notably, *in vitro* and *in vivo* analyses revealed a significantly enhanced binding affinity of pPB-MSCs for aHSCs compared to unmodified MSCs. The group also successfully demonstrated therapeutic efficacy in a murine model of liver fibrosis, indicated by marked [116] improvements in liver histopathology and a significant reduction in serum levels of hepatic injury biomarkers (alanine aminotransferase, aspartate aminotransferase, and alkaline phosphatase), attenuated extracellular matrix (ECM) deposition and reduce the population of α-SMA-positive aHSCs compared to unmodified MSCs, corroborating *in vitro* findings indicative of inhibited HSC activation. pPB-MSCs restored mitochondrial ultrastructure in fibrotic hepatocytes, suggesting a potential mechanism involving mitochondrial transfer. In other reported research both passive (Ultrasound mediated ECM degradation) and active targeting (Hyaluronic acid modification) were utilized to develop porous coordination network nanoparticles (PCN-NPs) remodel the ECM under ultrasound, enhancing drug access to activated hepatic stellate cells (aHSCs) delivering V-9302 (glutamine metabolism inhibitor) lead to resolution of fibrosis by disrupting hepatocyte-HSC pro-inflammatory crosstalk [117].

In another study [119], a multi-target approach, a nanocarrier composed of circular spherical DNA (cSNA) with surface decoration of circular PDGF-BB aptamer (a nuclease-resistant aptamer blocking PDGF-BB/PDGFR-β signal) and core composed of Collagenase I and ML290 (for reprogramming of pro-fibrogenic into pro-resolution macrophages by activating RXFP1 signalling). The cSNA releases collagenase I in fibrotic liver to degrade collagen and enhance nanoparticle penetration. The cSNA enables both upstream disease blockade and downstream tissue restoration, offering a novel therapeutic approach for fibrosis [119].

## Conclusion

Liver fibrosis has emerged as a major pathological outcome of various liver disorders, often progressing to hepatocellular carcinoma and contributing significantly to increased mortality, morbidity, and economic burden. Although numerous clinical trials have been conducted to address liver fibrosis associated with diverse etiologies, the majority were discontinued during Phase 2 or 3 due to a lack of therapeutic efficacy (*e.g.* Simtuzumab). Only a few drugs, such as Adafermin and Aramchol, have shown promising results in Phase 2 and 3 trials. Notably, Setanaxib is currently the only molecule approved by the U.S. FDA for the treatment of liver fibrosis.

In recent years, hepatic stellate cells (HSCs) have emerged as key mediators and therapeutic targets in liver fibrosis. Pharmacological interventions targeting liver fibrosis have demonstrated encouraging results in experimental models; however, clinical trials remain limited. Among these approaches, the active targeting of HSCs has shown significant promise in preclinical studies for the resolution of liver fibrosis. Combining active targeting strategies with antifibrotic agents in clinical trials may represent a promising avenue to enhance therapeutic success and clinical translation. Ultimately, such advancements aim to improve patient outcomes and overall quality of life.

## Future directions

HSCs have emerged as a promising and important target for treating liver fibrosis, leading to a complex yet promising area of research. HSCs play a central role in fibrogenesis after activation in response to liver insult, and express excess extracellular matrix proteins. However, potential challenges impede the development of effective therapies. Primarily, because HSCs share surface markers with other cell types, leading to potential off-target effects. Another challenge arises because HSCs are diverse cells that exhibit unique phenotypes depending on their location in the tissue and the disease state, which complicates the design and development of universal therapeutic options. Furthermore, the reversibility of fibrosis varies, with advanced stages posing greater resistance to therapeutic intervention. Additionally, efficient drug delivery to liver tissue is hindered by the liver’s complex anatomy and the presence of varying levels of the fibrotic matrix within the tissue. The major hurdle in the treatment monitoring aspect is the lack of reliable, non-invasive biomarkers for HSC activation, resulting in poor treatment monitoring. Moreover, the redundancy in fibrogenic signalling pathways leads to the fact that targeting a single molecular pathway often fails to halt disease progression. This is a crucial factor influencing the translation of promising experimental therapies into a clinical setting. This is evident by analysing the successful progression of clinical trials through various stages and regulatory approval for clinical use. Researchers are exploring ways to control HSC-immune cell interactions, restore HSC quiescence, or trigger their apoptosis to combat liver fibrosis. Artificial intelligence (AI) and computational tools are enhancing therapy design, while improved models like humanized mice and organ-on-chip systems are advancing drug testing. These strategies aim to develop more effective and targeted treatments.

To address these challenges, future research should focus on several innovative strategies, including precision delivery systems such as actively targeted nanocarriers loaded with more than one therapeutic molecule, nanoparticles, and antibody-drug conjugates, which are being developed to selectively target activated HSCs. Gene editing tools, such as CRISPR/Cas9 and RNA-based therapeutics, offer potential for modulating key fibrogenic genes and can be used to target multiple pathways, thereby mitigating liver fibrosis and its progression to liver cancer. Advances in single-cell transcriptomics and spatial omics studies can be helpful in two ways, as these studies can improve understanding of HSC heterogeneity, leading to a better understanding of unique phenotypes based upon location and tissue, paving the way for personalized treatments. Combination therapies that target multiple pathways or integrate anti-inflammatory and regenerative approaches may enhance efficacy and improve therapeutic outcomes. Additionally, modulating HSC-immune cell interactions and promoting either the reversion of HSCs to a quiescent state or their selective apoptosis are being explored. Computational modelling and AI will also be crucial for analysing data generated from single-cell sequencing data for the identification of potential novel targets, and a better understanding of the disease mechanism will also be an important aspect in optimizing therapeutic strategies.

In brief, future directions will be guided by advanced techniques, including single-cell transcriptome analysis and AI and machine learning, to identify novel therapeutic targets and options for treating liver fibrosis.
